# Conscience and conscientious objection: The midwife’s role in abortion services

**DOI:** 10.1177/0969733020928416

**Published:** 2020-07-06

**Authors:** Beate Ramsayer, Valerie Fleming

**Affiliations:** 151269 Liverpool John Moores University, UK

**Keywords:** Abortion, conscience, conscientious objection, midwifery

## Abstract

Traditionally, the role of midwives has been to be with women throughout the pregnancy continuum, from conception until the end of the postnatal period. Midwives, however, have been named as key providers of abortion services. While freedom of conscience is legally protected within Europe, discrepancies exist between midwifery and conscientious objection to abortion-related services. Midwives are largely ignored within the academic discussion despite the care and support they give to women undergoing abortions. Those discrepancies led to the aim of this article to address this issue by discussing some of the key ethical and legal concepts that are relevant to midwives’ role in the provision of abortion services.

This article shows that the decision to provide or object to abortion services remains ethically very complex because arguments exist both for and against its provision. Being with women can be interpreted differently and individual situations of care are multifaceted. Conscientious objection to abortion services is a highly contentious issue that has an overall importance to midwives. Noting that decisions are individual, may change or may be situationally dependant; a definitive position of midwives for or against conscientious objection cannot be assumed.

Respecting conscience and acknowledging that there are various arguments for and against conscientious objection promotes widespread understanding. It accommodates both the opportunity for midwives to object on conscience grounds to the provision of abortion services and respect women’s autonomy so that mutual agreement may be reached on issues that may have far reaching consequences.

## Introduction

The ethical issue of conscientious objection has been in existence for over a century, first coming to prominence in a 19th-century UK parliamentary debate concerning compulsory vaccination, where its naming was hotly challenged but ultimately included in the law.^1^ In this instance, it was parents rather than professionals who were allowed to exercise their consciences as to whether or not to have their children vaccinated.

Currently, a major subject of debate is that around the provision of abortion services and the related care of the women concerned. This debate is expanding internationally with both the provision of the service and health professionals’ rights to make an objection to its provision being highly contentious issues.^2,3^ Conscientious objection to providing abortion services describes the situation when health-care professionals decline to participate in the abortion process on conscience grounds.^4^ Many articles both for and against conscientious objection have been published in the academic literature.^2^ However, these have mainly been restricted to articles concerning the medical or legal professions with little attention being given to others such as midwives.

Despite midwives being designated by the World Health Organization as key providers of abortion services,^3^ and the legal protection afforded to freedom of conscience within Europe,^5^ few articles even mention them.^2^ However, legal and professional consequences for midwives, exercising a conscientious objection to participation in abortion services, have been documented throughout Europe,^6^ including some prominent legal cases that reached the highest courts in at least two countries, United Kingdom^7^ and Sweden.^8,9^ The aim of this article is thus to address this issue by discussing some of the key ethical and legal concepts that are relevant to midwifery practice. The role of the midwife is discussed first in order to contextualise the discussion.

## Expanding the scope of midwifery practice

Traditionally, the role of midwives has been to be with women throughout the pregnancy continuum, from conception until the end of the postnatal period.^10^ In some countries, this means that midwives hold the prime responsibility for women whose journeys are considered to be ‘normal’. Conversely, women with pre-existing pathologies, or who develop complications during the course of the journey, should always be the responsibility of a medical practitioner.^11^ However, in the latter case, midwives are still required to ‘be with’ women so that they may provide emotional and professional support even though they are not following a normal trajectory.

In the last few years, some midwives have developed specialist niches for themselves such as working with women who are drug or alcohol dependent, are refugees, are pregnant in early teenage years or are living in poverty. These midwives have advanced skills in their specific areas and, while being the main point of contact for the women concerned,^12^ are often working in close cooperation with senior medical practitioners.^11^ When the World Health Organization (WHO) named midwives as among the key providers of abortions,^3^ this appeared as another extension to the midwife’s traditional role. However, for a midwife to be with women undergoing abortions, a different set of skills is required because of the deliberate working towards the premature termination of the pregnancy. In this context, the term ‘being with women’ is seen as a dynamic, challenging and developing construct. The underlying professional philosophy means it is essential for a midwife to respect and support women in their individual needs and wishes during the childbirth continuum.^13^ Therefore, a midwife who chooses to embrace the expanded role of abortion provider, must be able to support the woman with ‘respectful maternity care’.^14^

For decades, it has been discussed that midwives take over sole responsibility for providing first trimester abortion services.^15^ In abortions taking place after the first trimester, midwives may be called upon to work with the women by administering the abortifacient drugs, accompanying the women during their labours, counselling women during their decision making processes or delivering the foetus and placenta.^16,17^ Their role, while physiologically paralleling that of an induced labour, involves the provision of a different kind of support. Qualitative findings suggest that women undergoing a second trimester abortion, have distinctive preferences and very specific emotional needs.^18^ This includes being ‘affirmed as moral decision-makers’, being ‘able to determine their degree of awareness during the abortion’ and having ‘care provided in a discrete manner to avoid being judged by others for having an abortion’.^19^

The above insights illustrate that ‘being with women’ undergoing a late abortion requires the midwife to support them in their individual situations. However, these needs can only be met by midwives who feel able to be with women in those situations. If midwives feel in conflict with their own conscience, they may not be a help to women undergoing abortions because of their emotional distance. It is such situations that give rise to claims of conscientious objection, which may be particularly relevant in relation to midwives’ respect for women’s needs. We discuss some of the ethical concepts from the literature that midwives confronted with such dilemmas may wish to consider.

## Conscience

### What is conscience?

The concept of conscience and conscientious objection is highly relevant within pluralistic societies and ethically complex situations, such as those existing in health care.^20^ As long ago as the thirteenth century, the theologian and philosopher Thomas Aquinas,^21^ focused one of his questions on conscience deducing that conscience determines that an act takes place in three ways: as witness that something was done or not done, as judgement as to whether something should or should not have been done or as an assessment of whether something was well or badly done.

It is the second of Aquinas’ principles that is highly relevant to midwifery practice in relation to abortions. In the context of the medical profession, Pellegrino^22^ referred to this stating that judgements of conscience are morally binding and must be followed, otherwise the person concerned would have behaved immorally. He also cautioned that while the application of conscience ‘may be in error about the facts, to ignore it may induce lasting feelings of guilt, remorse and shame’. Pellegrino further notes that both parties are entitled to respect for their personal autonomy as ‘all humans, ethicists included, possess an inner conviction of what is right and wrong and feel compelled to act in accord with that judgement’. Pellegrino’s explanations are equally relevant to midwives. Sepper^23^ acknowledged the multi-faceted nature of conscience, noting in the title *Not only the doctor’s dilemma* that conscience is not restricted solely to the medical profession. The importance of conscience is closely related to an individual’s moral integrity or sense of self and pointed out that, although some people are more conscientious than others, everyone has a conscience, thereby bringing its complexity to the fore. The seminal work of Wicclair,^24^ for example, provides a balanced overview between conscience and the duty to provide care. Wicclair’s work has underpinned other ethics discussions in law, philosophy and medicine because it proposes that the most promising ethical justification to conscientious objection is respect for moral integrity. However, some authors argue that the core ethical values on which decisions are based need to correspond with one or more core values in medicine.^25–27^ Wicclair’s work lacks consideration of the specific situations of midwives’ and other health professionals’ specific needs. However, Fleming et al.^2^ documented in their systematic review that midwives’ voices were notably absent.

Many commentators challenge the rights of health care professionals to allow their private values to interfere with their work.^28–30^ Others have proposed criteria for conscientious objection substituting responsibilities which they believe compensate for taking this stance.^31,32^

### Accommodating conscience

International protection has been afforded to conscience since the Universal Declaration of Human Rights of 1947.^33^ The European Convention of Human Rights, takes up the text in Article 9:

Everyone has the right to freedom of thought, conscience and religion…Freedom to manifest one’s religion or beliefs shall be subject only to such limitations as are prescribed by law and are necessary in a democratic society in the interests of public safety, for the protection of public order, health or morals, or the protection of the rights and freedoms of others.^5^

The Council of Europe later specified the right to conscientious objection to abortion in Resolution 1763:

No person, hospital or institution shall be coerced, held liable or discriminated against in any manner because of a refusal to perform, accommodate, assist or submit to an abortion, the performance of a human miscarriage, or euthanasia or any act which could cause the death of a human foetus or embryo, for any reason.^34^

The wording used by the Council is not restricted only to medical practitioners, and its clear statement is applicable to midwives and others, including institutions as a whole.

Wicclair^24^ recommended accommodating conscience but warned that there is no simple formula for so doing. Sepper^35^ agreed but took a broader perspective, noting that freedom of conscience is highly relevant for societies because of its links to freedom, tolerance and understanding.

While individuals may disagree over fundamental questions of morality, each person utilises their conscience in determining the morality of their own actions. The protection of conscience provides respect for various possible perspectives, which on the first view seem to be mutually incompatible. This was reiterated by Sulmasy^36^ who petitioned for tolerance, professional judgement and respect for the discretionary space of the physician. His view of conscientious objection to abortion in medicine proposed that abortion might be immoral for one person while it might be morally permissible for another stating:

Both ought humbly to acknowledge the fallibility of their positions and their inability to persuade each other of the truth of these positions, but this need not mean that one must conclude that neither position is true or that moral truth is subjective.

Widely diverging views have been articulated by Pellegrino^22^ who considered freedom of conscience as a moral right and Fiala and Arthur^37^ who classified conscientious objection as ‘dishonourable disobedience’ and as ‘unethical refusal to treat’. Furthermore, Liberman^38^ stated that conscientious refusals in health care can even be ‘moral wrongness’ and Pellegrino noted, ‘both the physician and the patient as human beings are entitled to respect for their personal autonomy. Neither one is empowered to override the other. The protection of freedom of conscience is owed to both’.^22^ Sepper,^35^ in keeping with Aquinas, added that it is the process of attempting to do what individuals perceive to be correct, rather than the outcome that provides a justification for protecting freedom of conscience. Addressing the complexity of conscientious objection in medicine, Sepper also advocated those involved should have the wisdom to defuse conflict situations by leaving space for conducting ethical conversations. This is something that still needs to be done in midwifery, as although the WHO has designated midwives as vital providers of the service, it also acknowledges midwives as second level health professionals.^3^ Unless in independent practice, midwives are afforded less opportunity to engage in such discussions as part of their normal working day. This matter is discussed at length by Thompson^39^ who acknowledges, however that she is considering the personhood of women rather than entering into the abortion debate.

Because the complexity of conscientious objection depends on moral principles as guiding decisions either to commit or omit a particular act, related decisions are, as elucidated 700 years ago by Aquinas, more than an application of rules.^35,40^ Harris^40^ spoke of a need for addressing not only conscientious objection within the debate but also ‘conscientious commitment’ that expresses the deliberate intentions of abortion-providers, Fovargue and Neal^41^ concurring but stressing that any objection must not address the individual woman but the treatment. From their perspective, each person’s own position must be open to differing conscience-based conclusions of others.

In the midwifery community, with its much higher numbers of practitioners than in medicine, it may be expected that wide ranging views will be held. In noting such a scenario, Sepper stressed that the complexity of the topic requires conscience to be taken seriously, noting that a health care provider might be willing to contribute in one circumstance and to refuse in another. While appearing to contradict the views of Fovargue and Neal, the focus here is perhaps on the grounds for abortion and is directly applicable to midwives. A midwife, for example, may be willing to provide the service if a woman has been subjected to rape or with serious foetal abnormalities but not for other reasons. These arguments are supplemented with an understanding that conscientious objection will remain an individual decision that can vary according to different situations as the topic is complex and controversial. Noting that decisions are individual, may change or may be situationally dependant, a definitive position of midwives for or against conscientious objection cannot be assumed. This means that midwives who decide to object to the provision of abortion services in some cases may argue differently in others by expressing a freedom of conscience within everyday professional life. Whatever stance an individual midwife may take, she will be bound by the laws of her country or professional codes of practice. These are discussed.

### Conscience clauses

Many countries have so called ‘conscience clauses’ enacted in law that offer some protection to health professionals, including midwives, who feel morally unable to provide abortion services on conscience grounds. Within most European countries, conscience clauses allowing health professionals to abstain from providing abortion related services are included in either specific legislation concerning abortion or employment. In Germany, for example, the law on conflicts in pregnancies states that no one is obliged to participate in abortion services,^42^ while the UK Abortion Act states ‘no person shall be under any duty, whether by contract or by any statutory or other legal requirement, to participate in any treatment authorised by this Act to which he has a conscientious objection’.^4^ The recently enacted law in the Republic of Ireland, rather than adopting a generalist approach, names specific health professionals, including midwives, in its clause on conscientious objection: ‘Nothing in this Act shall be construed as obliging any medical practitioner, nurse or midwife to carry out, or to participate in carrying out, a termination of pregnancy…to which he or she has a conscientious objection’.^43^

Other countries, such as Croatia, do not have a universal law. Instead the legislation on conscientious objection is linked to individual professions, with doctors being regulated by the Law on Medical Practice:

Because of their ethical, religious or moral belief or beliefs, doctors have the right to assert a conscientious objection and refuse to conduct diagnosis, treatment and rehabilitation of the patient, if doing so does not conflict with the rules of the profession, and if it does not cause permanent damage to the health of or threaten the life of a patient.^44^

The Nursing Act allows conscientious objection for nurses:

Because of their ethical, religious or moral belief or beliefs, nurses have the right to assert conscientious objection and refuse to conduct medical/nursing care if doing so does not conflict with the rules of the profession, and if it does not cause permanent damage to the patient’s health or endanger the patient’s life.^45,46^

As yet, however, there is no specific midwifery legislation meaning that a midwife may be forced into providing abortion services against her own conscience such as was the case with Jaga Stojak.^6^ This is different from France, for example, where: ‘No midwife is obliged to participate in a voluntary abortion’.^47^ Each of these laws has in common that, unless there is a critical risk of death for the woman, no one can be forced to provide abortion services. On this legal level, the debate concerning conscientious objection is not about ethical issues but generally bringing the European legislation to country level. The legislation may be backed up by professional codes of practice or, where there is no legislation, these codes may provide the only guidelines available.

### Professional codes of practice

Several professional codes of practice exist, which vary in their position and supporting arguments. Here, midwives are not well served as, despite the WHO’s reference to midwives as key providers of abortion services,^3^ most well-developed professional codes of practice are applicable to the medical profession and have been drafted by their various professional bodies. The International Federation of Obstetricians and Gynaecologists’ resolution on conscientious objection acknowledged that practitioners ‘have a right to respect for their conscientious convictions both not to undertake and to undertake the delivery of lawful services’^48^ and their position statement on conscientious objection to abortion further specified: ‘Practitioners have a right to respect for their conscientious convictions in regard to both undertaking and not undertaking the delivery of lawful procedures, and not to suffer discrimination on the basis of their convictions’.^49^

However, midwifery’s comparable organisation, the International Confederation of Midwives (ICM), appears to ignore the issue despite the latest update of its essential competencies for midwifery practice.^50^ In this document, competency 1e states ‘[midwives must] uphold fundamental human rights when providing midwifery care’ and listed under this is knowledge of law. Similarly, competency 2i declares that a midwife must ‘provide care to women with unintended or mistimed pregnancy’ wherein a midwife is expected to have knowledge of options for abortion and the use of appropriate medications as well as the risks of unsafe abortion. In addition, ‘midwives who have to implement certain skills’ are to

prescribe, dispense, furnish or administer drugs according to the scope of practice and protocol (however authorized to do so in the jurisdiction of practice in dosages appropriate to induce medication abortion) and perform manual vacuum aspiration of the uterus up to 12 completed weeks of pregnancy.

The ICM clearly supports the drive by WHO to cascade the role of primary provider of abortion services from doctors to midwives. However, it is somewhat unexpected that a statement on conscientious objection has not been published by midwifery’s international body. This is even more surprising when the modified Delphi Study, on which the ICM competency on abortion is based, is considered. This study, administered via the Internet, involved three rounds of data collection where 37 statements of abortion-related knowledge and skill were presented. In addition, in-depth review of the literature was conducted that focussed on relationship between maternal mortality and morbidity and access to safe abortion. This literature review outlined a strong relation between global access to abortion and global maternal morbidity and mortality rates.^51^

They reaffirmed the role of midwives in the provision of abortion-related services, including the provision of medical abortion, performance of vacuum aspiration, comprehensive counselling, referral for abortion, the provision of post-abortion services and post abortion family planning. While the authors respected conscientious objection as a possible stance of midwives who decided not to provide those services, this finding from their study has not been translated into the ICMs’ essential competencies.

At country level, midwives are often also not clearly advised with regard to their legal rights. The Royal College of Midwives in the United Kingdom makes it clear that all midwives should be prepared to care for women undergoing abortions.^52^ It briefly states that the conscience clause in the 1967 Abortion Act allows them the right to object, cautioning that ‘this should only apply to direct involvement in the procedure of terminating a pregnancy’. What constitutes ‘direct involvement’ is, however, far from clear despite a ruling from the UK Supreme Court on a case concerning two midwives who held long-standing positions as conscientious objectors and who held senior roles in the delivery unit of a hospital where mid trimester abortions were carried out on a daily basis.^7^ In their findings, the judges ruled that the only part of their role to be covered fully by the conscience clause, was being present to assist and support if medical intervention were required.

The Northern Ireland Office has recently legalised abortion in line with the rest of the United Kingdom but is currently in an ‘interim period’ during which consultation is being made on a variety of issues including conscientious objection. During the consultation guidelines include those of the General Medical Council and Nursing and Midwifery Council. This guidance is interesting because it respects and includes some of the ethics based literature and it becomes clearer how conscientious objection could be applied to practice.^53^ Those thoughts greatly impact on the present debate on conscientious objection and in particular, how they are translated into practice by midwives. In Germany, for example, some midwives have recently stated they have no choice because they feel under pressure to provide abortion services as this has been delineated as a clear midwifery task.^16^ This is astonishing because as shown above, a conscience clause does exist in Germany and it seems as if midwives are not aware of it. It thereby calls into question the motives of those who claim that there is no choice and outlines lack of guidance by their professional associations. Midwives who may wish to make a conscientious objection to participation in abortions services thus do not always appear to have support from their professional organisations.

## Conclusion

Conscientious objection has an overall importance to midwives which means that the midwife’s decision either to provide or to object to the provision of abortion services should be respected. The above insights offer understanding for different possible stances that midwives may decide to choose during their decision-making processes when abortion services are requested. Different arguments are available for or against conscientious objection, each of which contribute to an understanding that midwives can choose different stances either to provide or to object to providing abortion services. This should occur without the professional sanctions that have happened in the past because the main task of midwives is care provision during normal processes during the childbirth continuum.

It is critical to note, that midwives have been designated as main providers of abortion services because both the process of abortion and the provision of emotional care differs completely from that required in the physiological processes of normal birth. It seems as if this has come about as a means to overcome and cover personnel shortages in the medical sector. The ICM’s rapid adjusting of their previously research based ‘Essential Competencies for Midwives’ has further contributed to the aforementioned feelings of coercion. For midwives to feel valued and included they need to be called upon to contribute to further discussions.

Conscientious objection to the provision of abortion services is important for midwives because it respects midwives’ internal values and the espoused values of the profession. An ongoing debate is needed within the complex field of midwifery and conscientious objection to abortion services as to how this can be realised in practice according to the law. Women seeking abortion-services may also benefit from conscientious objection because this ensures that they will be cared for by midwives who consciously provide the necessary emotional and physical care needed. Respecting conscience and acknowledging that there are various arguments for and against conscientious objection develops an understanding, that both accommodates the opportunity for midwives to object on conscience grounds to the provision of abortion services and respect women’s autonomy so that mutual agreement can be reached on issues that may have far reaching consequences.
